# Supplementary material for: ROS/TNF-α Crosstalk Triggers the Expression of IL-8 and MCP-1 in Human Monocytic THP-1 Cells via the NF-κB and ERK1/2 Mediated Signaling
Source: Int J Mol Sci. 2021 Sep 29;22(19):10519. doi: 10.3390/ijms221910519 (PMC8508672; doi:10.3390/ijms221910519)
Supplement: Supplementary file 1 [file ijms-22-10519-s001.zip › ijms-1366438-supplementary.pdf]

Table S1. Clinico-demographic data of the study participants

Parameter	Lean	Overweight	Obese
Age (Yrs)	41.63 ± 10.38	45.80 ± 9.10	47.73 ± 14.36
Gender (M/F)	7/8	5/10	10/5
BMI (kg/m²)	22.86 ± 1.89	27.80 ± 1.60	35.08 ± 3.73
PBF (%)	26.96 ± 4.62	34.81 ± 4.38	38.31 ± 3.96
FBG (mmol/L)	4.95 ± 0.63	5.08 ± 0.49	5.72 ± 1.65
Cholesterol (mmol/L)	5.25 ± 0.98	5.27 ± 0.90	4.85 ± 1.34
TGL (mmol/L)	0.85 ± 0.45	1.28 ± 0.56	1.32 ± 0.85
LDL (mmol/L)	3.34 ± 0.79	3.41 ± 0.78	3.09 ± 1.03
HDL (mmol/L)	1.52 ± 0.49	1.32 ± 0.39	1.15 ± 0.24
HbA1c (%)	5.56 ± 0.42	5.60 ± 0.42	6.10 ± 1.39

PBF: Percent body fat; FBG: Fasting blood glucose; TGL: Triglycerides; LDL: Low-density lipoprotein; HDL: High-density lipoprotein; HbA1c: Glycated hemoglobin (hemoglobin A1c). All data, except gender (male to female ratio) represent mean ± SD values.

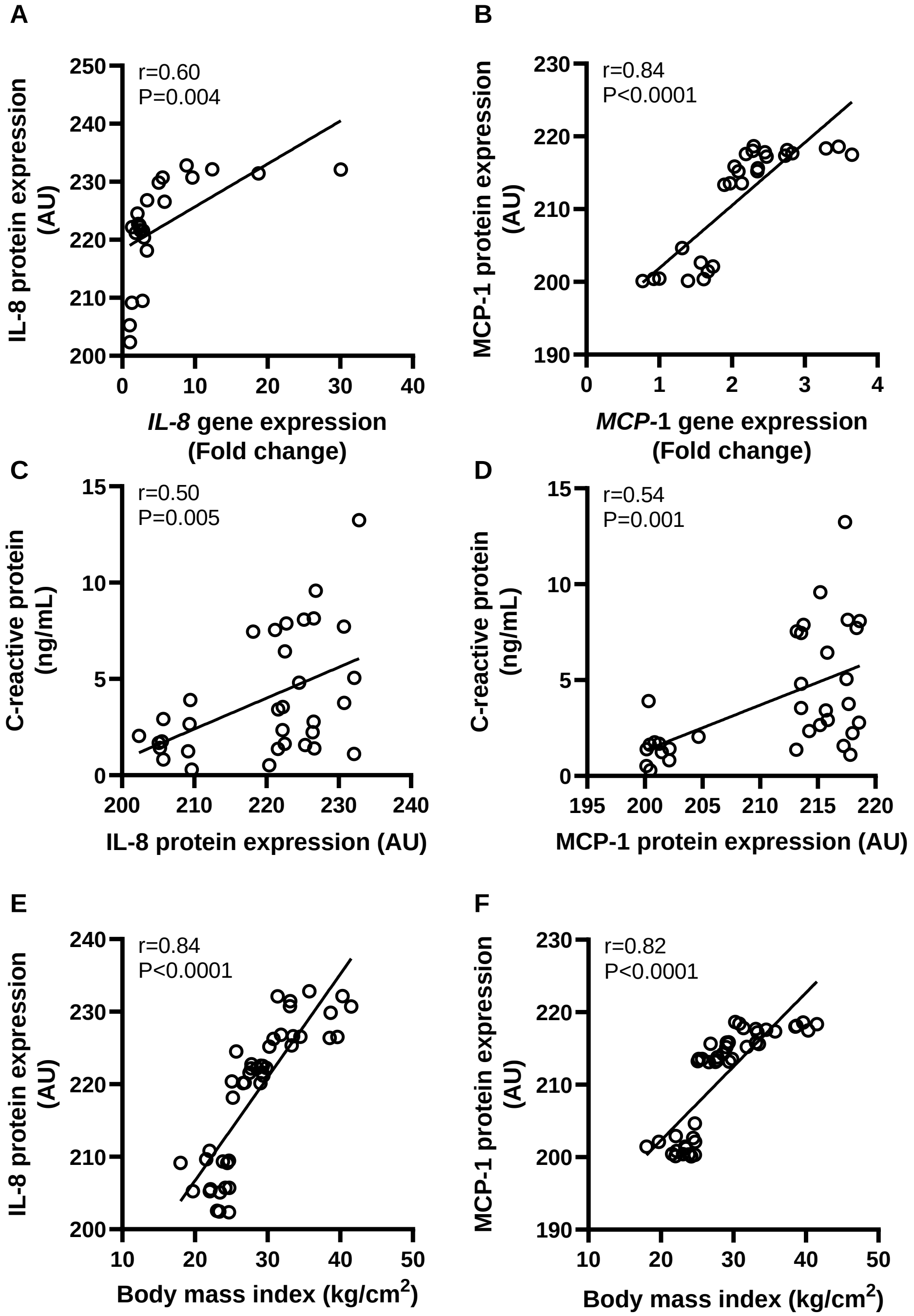

Figure S1. Correlation of adipose IL-8 and MCP-1 protein expression with respective gene expression, C-reactive protein (CRP) levels and body mass index (BMI). IL-8 and MCP-1 protein expression was determined in adipose tissue (AT) samples, 15 each, from lean (BMI: 22.86±1.89 kg/cm^2), overweight (BMI: 27.80±1.60 kg/cm^2), and obese (BMI: 35.08±3.73 kg/cm^2) individuals using immunohistochemistry (IHC), expressed as arbitrary units (AU). Total RNA collected from adipose tissue samples was used to assess the mRNA expression of *IL-8* and *MCP-1* (fold change) using qRT-PCR. Plasma high sensitivity (hs) CRP levels were measured by using ELISA (Biovendor LLC, Asheville, NC, USA), following the manufacturer's instruction. The data obtained from 3 independent determinations with similar results show that IL-8 and MCP-1 protein expression associated positively with: (A, B) *IL-8* mRNA (P=0.004) and *MCP-1* mRNA (P<0.0001) levels, respectively; (C, D) CRP levels (IL-8: P=0.005, MCP-1: P=0.001); and (E, F) BMI levels (IL-8: P<0.0001, MCP-1: P<0.0001).

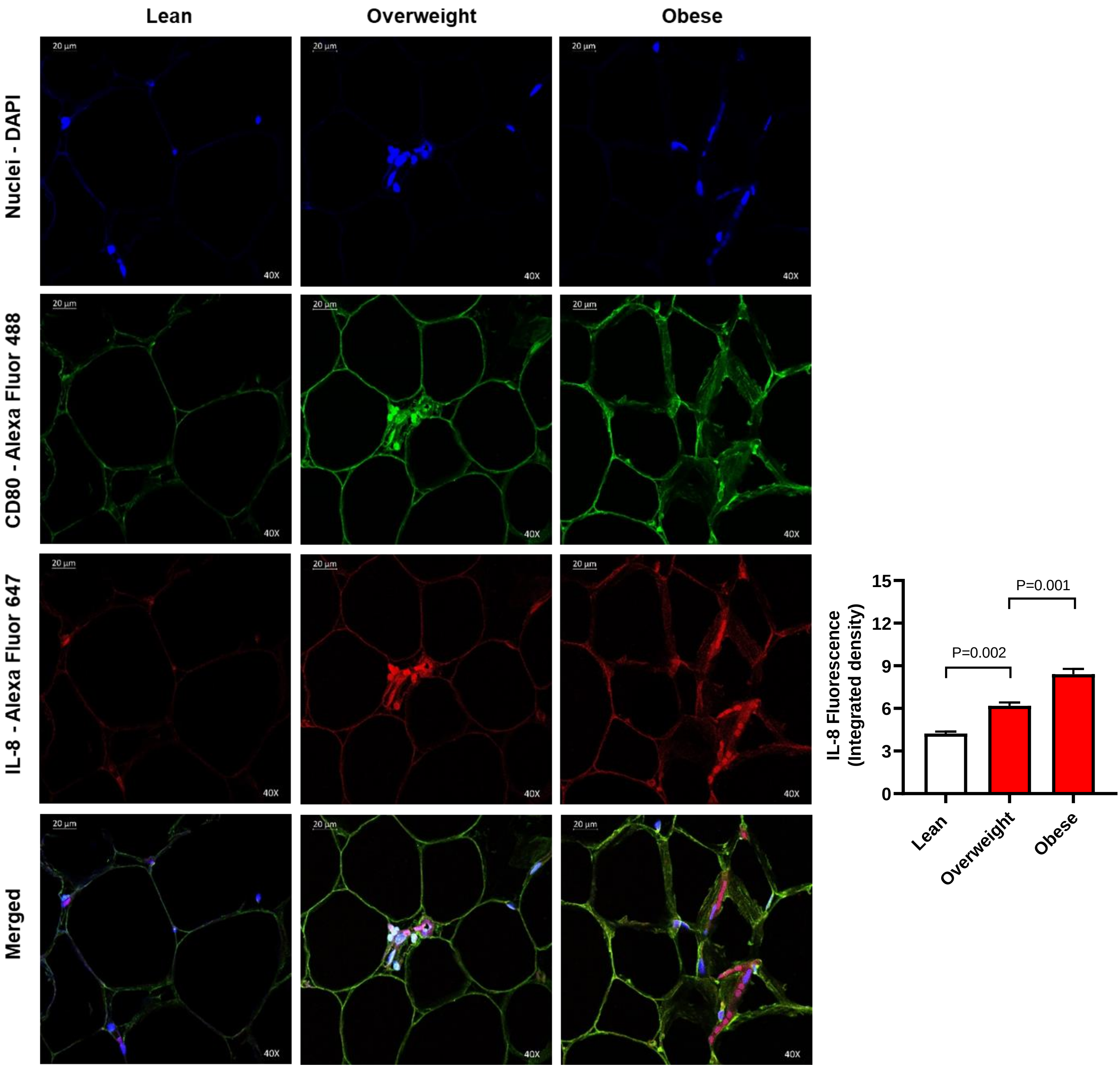

Figure S2. Adipose tissue IL-8 expression in lean, overweight, and obese individuals. IL-8 protein expression was detected in inflammatory monocytes/macrophages in the adipose tissue samples from lean (BMI: 22.84±2.74 kg/cm^2), overweight (BMI: 28.53±1.66 kg/cm^2), and obese (BMI: 34.60±4.29 kg/cm^2) individuals, 4 each, using confocal microscopy. Briefly, after antigen retrieval and blocking, samples were incubated for 2h with anti-human CD80 mouse mAb (1:200 dilution, MA5-15512, ThermoFisher Scientific). After washing, samples were incubated overnight at RT with rabbit polyclonal anti-human IL-8 primary Ab (1:200 dilution, ab106350, Abcam). After 3 washes, samples were incubated for 1h with secondary Abs; first with AF488-conjugated goat anti-mouse (1:400 dilution, ab150113, Abcam), and after 3 washes with AF647-conjugated goat anti-rabbit (1:400 dilution, ab150079, Abcam). Samples were counterstained with DAPI, mounted and analyzed by confocal microscopy as described in Methods. IL-8 fluorescence intensity was detected in 10 fields of each image and mean integrated density was calculated for each image. The representative images (40× magnification) from 3 independent stainings with similar results show the increased IL-8 expression in obese and overweight as compared to lean adipose tissue. Group differences were calculated using one-way ANOVA (Tukey's multiple comparisons test).

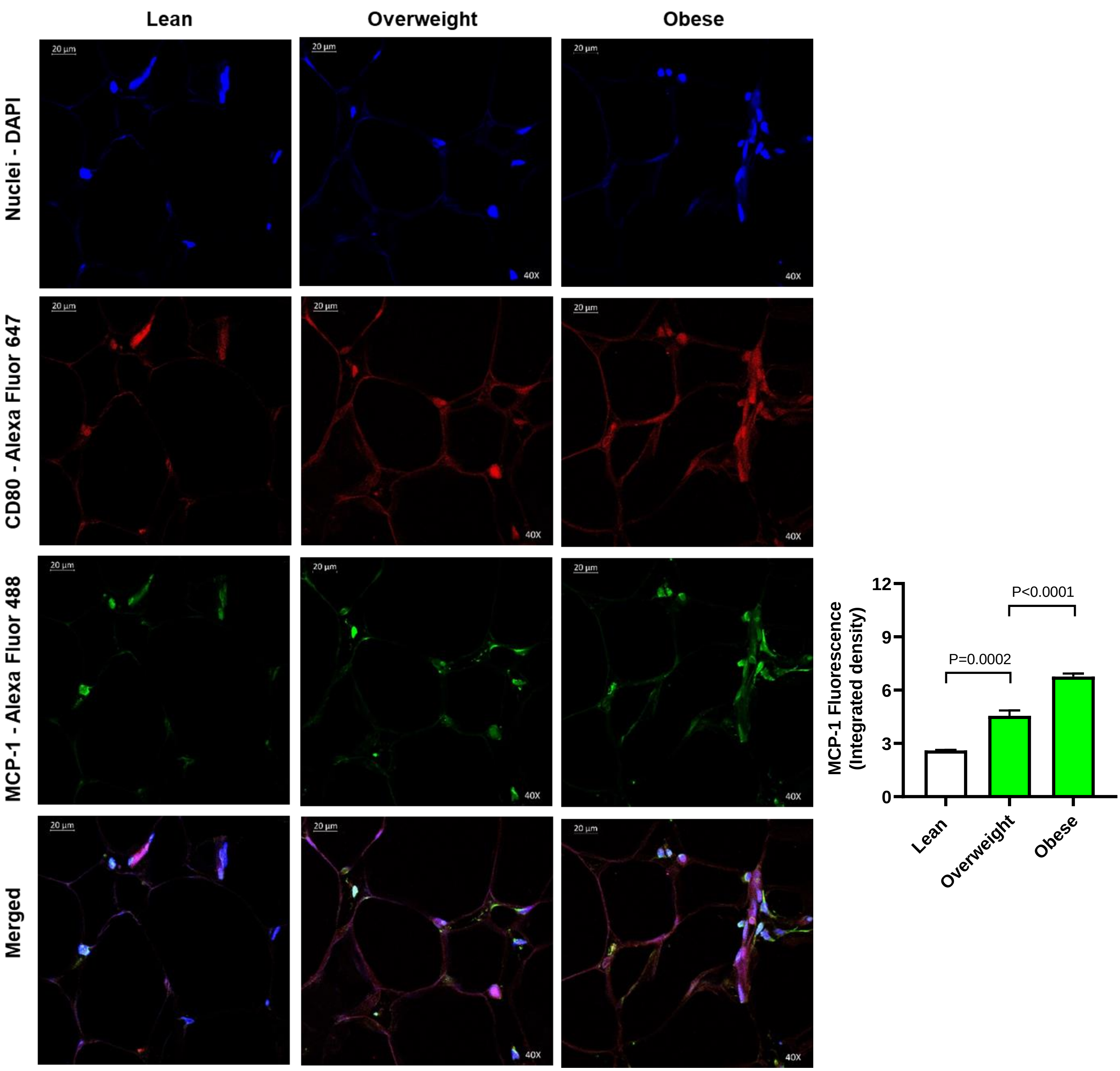

Figure S3. Adipose tissue MCP-1 expression in lean, overweight, and obese individuals. MCP-1 protein expression was detected in monocytes/macrophages in the adipose tissue samples from lean (BMI: 22.84±2.74 kg/cm^2), overweight (BMI: 28.53±1.66 kg/cm^2), and obese (BMI: 34.60±4.29 kg/cm^2) individuals, 4 each, using confocal microscopy. Briefly, after antigen retrieval and blocking, samples were incubated for 2h with anti-hu CD80 mouse mAb (1:200 dilution, MA5-15512, ThermoFisher Scientific). After washing, samples were incubated overnight at RT with rabbit polyclonal anti-human MCP-1 primary Ab (1:100 dilution, PA5-80413, ThermoFisher Scientific). After 3 washes, samples were incubated for 1h with secondary Abs; first with AF647-conjugated goat anti-mouse (1:400 dilution, ab150115, Abcam), and after 3 washes with AF488-conjugated goat anti-rabbit (1:400 dilution, ab150077, Abcam). Samples were counterstained with DAPI, mounted and analyzed by confocal microscopy as described in Methods. MCP-1 fluorescence intensity was detected in 10 fields of each image and mean integrated density was calculated for each image. The representative images (40× magnification) from 3 independent stainings with similar results show the increased MCP-1 expression in obese and overweight as compared to lean adipose tissue. Group differences were calculated using one-way ANOVA (Tukey's multiple comparisons test).

A

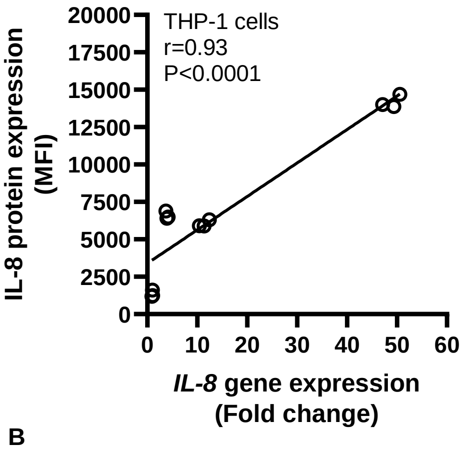

B

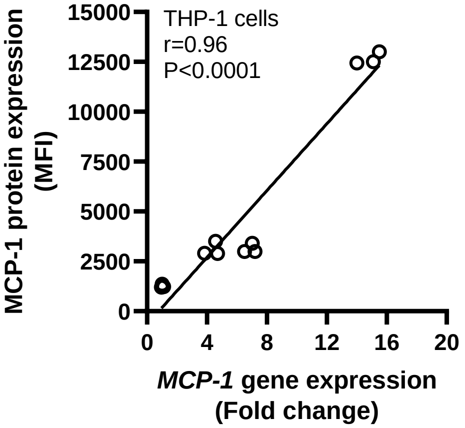

Figure S4. Association between gene and protein expression of IL-8 and MCP-1. THP-1 cells were stimulated, in triplicate, with TNF-α (10 ng/mL) and/or H_2O_2 (10 mM) for 24h, while control cells were treated with vehicle only. IL-8 and MCP-1 gene expression (fold change) and protein expression (MFI) was determined by qRT-PCR and flow cytometry, respectively, as described in materials and methods. The data (mean±SEM) obtained from three independent experiments with similar results show strong positive association between the gene and the protein expression of (A) IL-8 ($r=0.93$, $P<0.0001$) and (B) MCP-1 ($r=0.96$, $P<0.0001$).

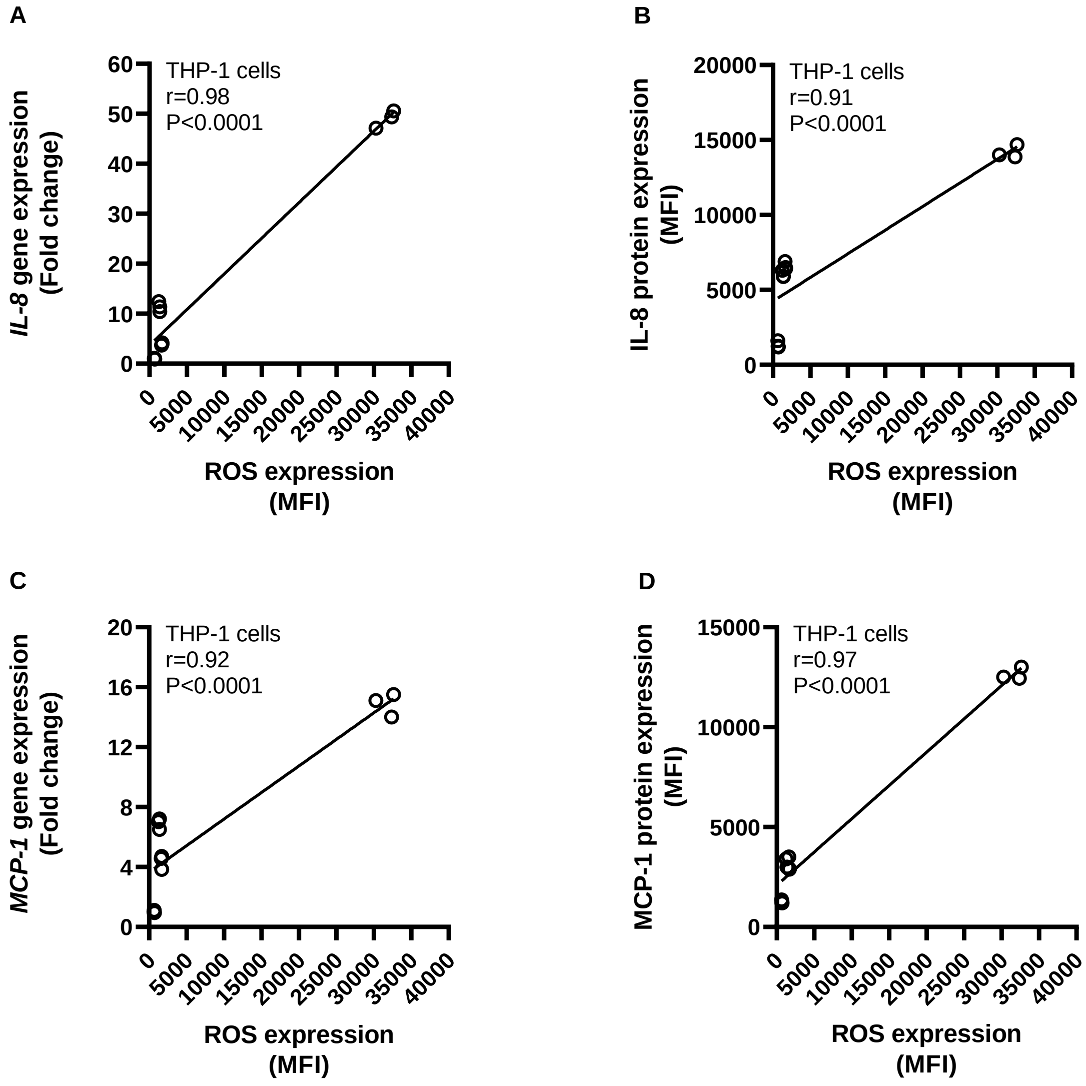

Figure S5. Association between ROS and IL-8/MCP-1 gene and protein expression. THP-1 cells were stimulated, in triplicate, with TNF-α (10 ng/mL) and/or H_2O_2 (10 mM), while control cells were treated with vehicle only. ROS expression (MFI) was detected using DCFH-DA assay and DCF was measured by flow cytometry, while IL-8/MCP-1 transcripts and protein levels were measured by using qRT-PCR and flow cytometry, respectively. The data (mean±SEM) obtained from three independent determinations with similar results show strong positive correlation (P<0.0001) between ROS and levels of (A) *IL-8* mRNA expression (r=0.98), (B) IL-8 protein expression (r=0.91), (C) *MCP-1* mRNA expression (r=0.92), and (D) MCP-1 protein expression (r=0.97).

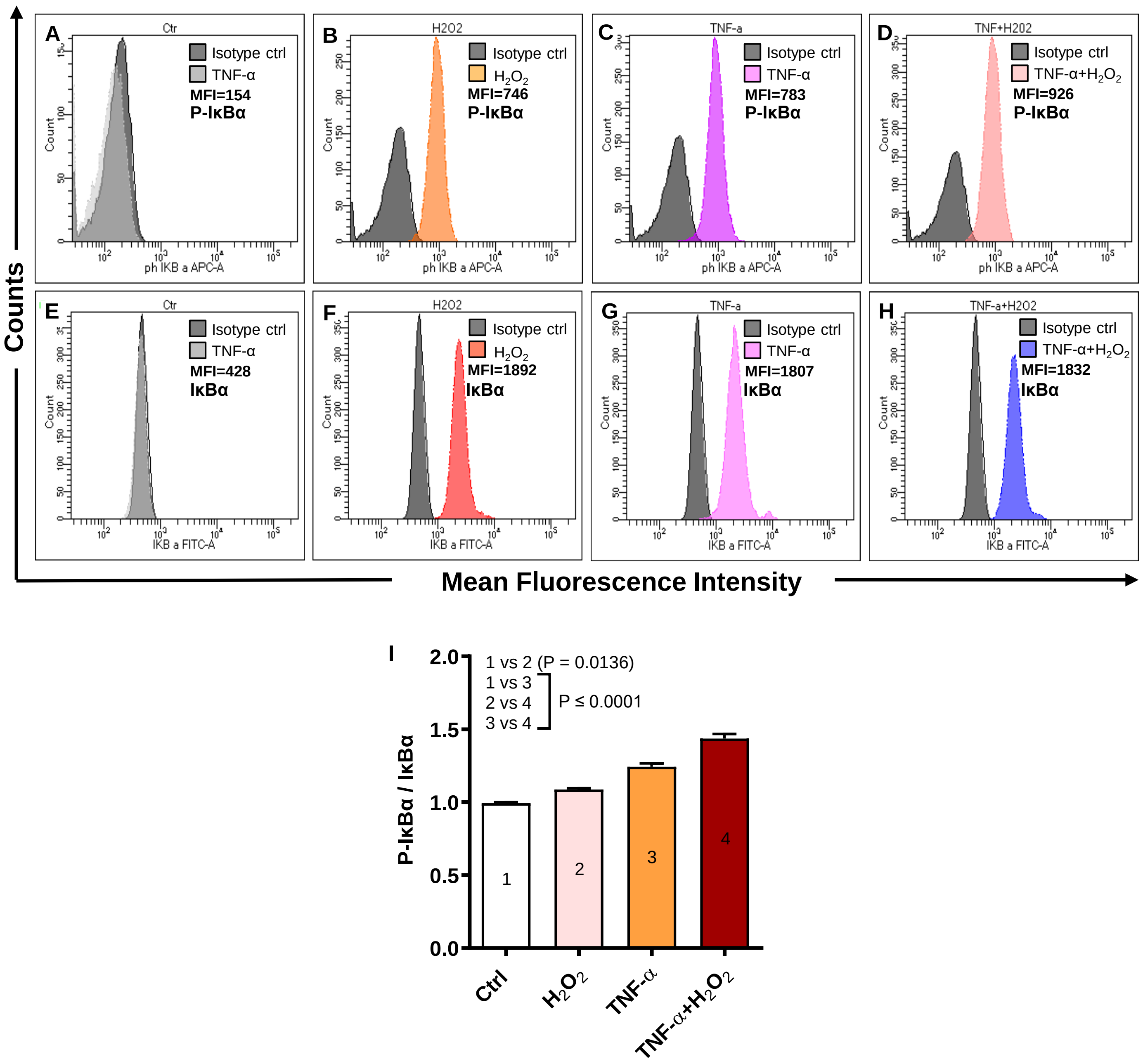

Figure S6. Ratio of phosphorylated to total IκB*α* expression. THP-1 cells were stimulated, in triplicate wells, with TNF-*α* (10 ng/mL, for 5 min) and/or H_2O_2 (10 mM, for 15 min), while controls were treated with vehicle only. Expression of phosphorylated and total IκB*α* was assessed using flow cytometry. Briefly, cells were washed with permeabilization buffer and stained using, 5μL each, of APC-conjugated anti-phospho (Ser32/Ser36)-IκB*α* (Cell Signaling, USA) and FITC-conjugated IκB*α* (BD Biosciences, USA) mouse mAbs for 20 min in fixation/permeabilization solution. Cells were washed with permeabilization buffer and resuspended in PBS containing 2% paraformaldehyde for FACS analysis. Representative histograms show expression (MFI) of: (A-D) phosphorylated and (E-H) total IκB*α*. Group differences were calculated using one-way ANOVA (Tukey's multiple comparisons test). The data (mean±SEM) obtained from three independent experiments with similar results show significant increase in phosphorylated to total IκB*α* ratio in THP-1 monocytic cells, following treatments with vehicle (Ctrl), H_2O_2, TNF-*α*, and TNF-*α*+H_2O_2, respectively.
